# Estimating the Costs and Benefits of Providing Free Public Transit Passes to Students in Los Angeles County: Lessons Learned in Applying a Health Lens to Decision-Making

**DOI:** 10.3390/ijerph111111384

**Published:** 2014-10-31

**Authors:** Lauren N. Gase, Tony Kuo, Steven Teutsch, Jonathan E. Fielding

**Affiliations:** 1 Division of Chronic Disease and Injury Prevention, Los Angeles County Department of Public Health, 3530 Wilshire Blvd, 8th floor, Los Angeles, CA 90010, USA; E-Mail: tkuo@ph.lacounty.gov; 2 David Geffen School of Medicine, University of California, Los Angeles, 10880 Wilshire Blvd, Ste. 1800, Los Angeles, CA 90024, USA; 3 Los Angeles County Department of Public Health, 313 N Figueroa St., Los Angeles, CA 90012, USA; E-Mail: steventeutsch@gmail.com; 4 Fielding School of Public Health, University of California, Los Angeles, 640 Charles E Young Dr., Los Angeles, CA 90095, USA; E-Mail: jfieldin@ucla.edu

**Keywords:** health impact assessment, health in all policies, public transportation, education, youth

## Abstract

In spite of increased focus by public health to engage and work with non-health sector partners to improve the health of the general as well as special populations, only a paucity of studies have described and disseminated emerging lessons and promising practices that can be used to undertake this work. This article describes the process used to conduct a Health Impact Assessment of a proposal to provide free public transportation passes to students in Los Angeles County. This illustrative case example describes opportunities and challenges encountered in working with an array of cross-sector partners and highlights four important lessons learned: (1) the benefits and challenges associated with broad conceptualization of public issues; (2) the need for more comprehensive, longitudinal data systems and dynamic simulation models to inform decision-making; (3) the importance of having a comprehensive policy assessment strategy that considers health impacts as well as costs and feasibility; and (4) the need for additional efforts to delineate the interconnectivity between health and other agency priorities. As public health advances cross-sector work in the community, further development of these priorities will help advance meaningful collaboration among all partners.

## 1. Introduction

There is increasing recognition among public health practitioners and researchers that health is influenced by many factors outside the direct control of public health and the healthcare system. To improve the conditions where people live, work, learn and play, public health leaders are starting to place greater emphasis on working with non-health sector partners, including transportation authorities, planners, educators, and officials from the justice system [1,2]. The term “Health in All Policies” (HiAP) has been used to describe such efforts aimed at improving health by incorporating health considerations into decision-making across policy areas [3].

While there are many ways to operationalize HiAP, structured process and tools can help guide implementation; Health Impact Assessment (HIA) is one such tool which can be used to incorporate health considerations into decision-making [4,5]. HIA is defined by the Institute of Medicine (IOM) as “a systematic process that uses an array of data sources and analytic methods and considers input from stakeholders to determine the potential effects of a proposed policy, plan, program or project on the health of a population and the distribution of the effects within the population” [6]. The analytic assessment traditionally follows a six-step framework: screening, scoping, assessment, recommendations, reporting, and monitoring and evaluation [6]. A recent national evaluation found that HIAs can contribute meaningfully to the decision-making process, helping to achieve policy outcomes that promote health [7].

The expanded role of public health practitioners in influencing decisions made in other sectors is likely to bring a new set of opportunities and challenges. However, despite the increased proliferation of HiAP and HIA projects in the United States, only a paucity of studies have described and disseminated emerging lessons and promising practices that can be used to undertake this work [3,8]. For example, few case studies have shed light on ways in which HiAP has been operationalized at the local level or highlight the opportunities for advancing and aligning local with national efforts.

The purpose of this article is to present such a case study, describing the process used to conduct a HIA of a proposal to provide free public transportation passes to students in Los Angeles County (LAC), focusing specifically on identifying the challenges and lessons learned in working with a wide array of cross-sector partners. We begin with an overview of the methods used to conduct the HIA and the major findings and recommendations from the assessment. We then discuss four major lessons learned from our efforts to engage non-health sector partners, highlighting both the utility of the HIA to informing ongoing policy dialogue and challenges to moving HiAP and HIA work forward.

## 2. Methods of the Health Impact Assessment

### 2.1. Proposal Background

Although many students cite lack of convenient, affordable transportation as a barrier to school attendance [9], most school districts in LAC do not provide school bus service to students [10]. In April 2013, the Los Angeles County Education Coordinating Council (ECC) adopted a resolution to address this, calling for the Council to “collaborate with school districts, other organizations, and the Los Angeles County Metropolitan Transportation Authority (MTA) to secure free Metro (“transit”) passes for all students from preschool to college,” regardless of income [11]. The ECC, a collaborative effort of stakeholders across LAC, serves as an advisory body to the County of Los Angeles government’s Chief Executive Office. Its membership includes leaders and decision-makers from school districts, county departments, the juvenile court, the county children’s commission, advocacy and planning groups, community agencies, alumni youth and caregivers [12]. The MTA represents the largest bus and rail transportation provider in the southern California region. The transit pass resolution recommends providing free bus and rail passes that can be used 24 h a day, 7 days a week by all students across this jurisdiction.

At the time of the resolution’s adoption, limited research was available to support the potentially controversial proposal. A paucity of information, for example, was available about the extent to which school attendance or fare evasion citations could be impacted through such expanded coverage of youth ridership. Other potential benefits, such as increased access to after-school programming, were also not well characterized, nor were the potential costs of the program.

The Los Angeles County Department of Public Health (DPH) partnered with a sub-committee of the ECC, the School Attendance Task Force (SATF), in May 2013 to initiate a HIA on the transit pass proposal. DPH determined that a HIA could add value because: (a) the proposal had the potential to impact health, yet health was not being considered; (b) existing data were available, but had not been synthesized; and (c) decision makers were eager to better understand the proposal’s potential costs and benefits. To align with ongoing dialogue, which accelerated upon passage of the resolution in April 2013, the HIA was completed in October 2013 [13].

### 2.2. HIA Scope and Methods

**Figure 1:**
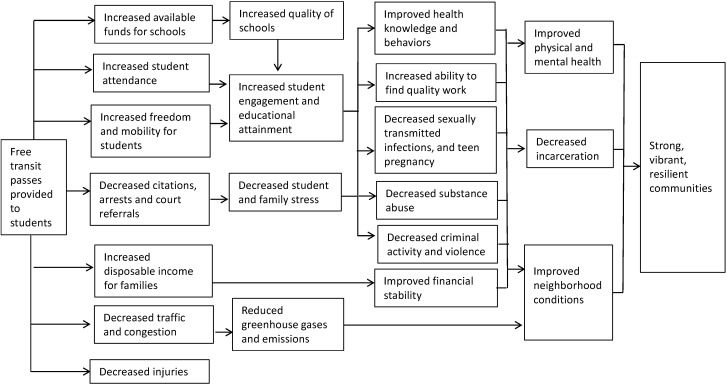
Potential benefits of providing free public transit passes to students, pathway diagram, Los Angeles, California ^1^.

^1^ This pathway diagram was developed during the scoping stage of the Health Impact Assessment (HIA) based on conversations with HIA stakeholders and subject matter experts. The diagram was iteratively refined during the assessment phase based on information gathered from literature reviews, key informant interviews, and analyses of secondary data sources. For more details of the results upon which this diagram is based, see the full HIA report [13].

The HIA defined the policy options as described in the ECC proposal: the universal provision of bus and rail passes for all students in kindergarten through college, without any income or time of day restrictions. Although not specified in the proposal, in order to illustrate trade-offs, alternative scenarios were considering during the assessment, including providing passes only to: (a) elementary, secondary, or college students; and (b) low-income students. Since the major goals of the ECC resolution were to improve school attendance and reduce fare evasion citations issued to youth, these impacts—along with their associated health outcomes—were the central focus of the HIA. Additional potential benefits included traffic volume and congestion, injuries, opportunities for physical activity, available funds for schools, disposable income for families, and freedom and mobility for youth (Figure 1). SATF members expressed a desire to better understand the potential cost consequences of the proposal, including changes in fare revenues and transit ridership. Thus, DPH estimated these costs as part of the HIA.

A diverse array of stakeholders were engaged throughout all phases of the HIA to define the topic and scope, provide input on the assessment questions and data sources, review draft products, and disseminate the results. Major stakeholder groups that participated in this process included the MTA, other County departments and agencies (e.g., Probation, Sheriff), school districts (which are independent of County government), the Los Angeles juvenile courts, and community-based organizations [13].

Mixed qualitative, quantitative, and economic analytic methods were used to complete the HIA. First, we conducted a review of the published literature on the costs and benefits of free or discounted transit pass programs using a variety of databases (e.g., Google Scholar, PubMed, ERIC) and websites of HIA stakeholders and government agencies, including the MTA, the Los Angeles Unified School District (LAUSD), the U.S. Census Bureau, and the Environmental Protection Agency. Searches were conducted based on each of the cost/benefit categories (e.g., the connection between transportation and school attendance, relationship between public transit use and physical activity levels in youth). To be included, sources had to be peer-reviewed or published by a credible source. Sources that were published within the last 20 years and described work conducted within the U.S. were prioritized.

Second, we consulted experts in the field of transportation and education. Experts were identified through relationships with the SATF and internet searches to identify jurisdictions that had implemented a free or reduced-cost transit program for youth. In total, we conducted nine key informant interviews with individuals from LAC Sheriff’s Department, LAUSD, MTA, Boston Public Schools (Boston, MA, USA), Urban Habitat (San Francisco, CA, USA), Organizing People, Active Leaders (Portland, OR, USA), and the Mid-City Community Advocacy Network (San Diego, CA, USA).

Third, we compiled and conducted analyses of existing data, including: (a) MTA administrative data from fiscal year 2013 to estimate revenue received from youth taking advantage of student pricing; (b) the 2001 and 2011 Southern California Association of Governments Household Travel Survey, which collects information on travel behavior by members of a random sample of households in Southern California, to estimate average daily rates of public transit use and fares paid; (c) the 2011 MTA On-Board Survey to estimate characteristics of MTA public transit users; (d) LAUSD (the region’s largest school district, with over 640,000 students) administrative records on the number of unexcused absences in the 2012–2013 school year; and (e) LAC Sheriff administrative records on the number and types of youth who received fare evasion citations in 2012. In order to completed the HIA within a timeline that could help inform decision-making, no primary data collection was conducted. Because no primary data was collected, Institutional Review Board approval was not necessary. In addition to the full report, which was released in October 2013, DPH produced an *Addendum*, released in April 2014, highlighting a selected number of updated estimates and additional data points from a field poll survey of LAC residents [14].

## 3. Major Findings and Recommendations from the Health Impact Assessment

### 3.1. Potential Costs

Providing free transit passes to all students in LAC could result in significant costs to transportation agencies in the region. Estimates from MTA suggest that students contributed over $20 million in fare revenues in 2013. The overall costs are likely to be even larger because this estimate (a) does not include losses to other LAC transit operators which represent at least 15% of the total transit market share in the jurisdiction and (b) only includes students who take advantage of student pricing. An estimate of potential revenue losses using average daily rates of use of public transit and fares paid by students from the Southern California Association of Governments suggests that universal provisioning of transit passes to students could result in more than a one-fifth decrease in transit fare revenues (Table 1). Based on MTA fare revenues ($340 in fiscal year 2013), this could equate to a loss of $71 million with a pass for all students. Under the alternative scenario of proving passes only to students living in households below the 2011 federal poverty guidelines, the expected costs were much lower—a universal provision of transit passes was estimated to result in a 7% decrease in transit fare revenue [14].

Using published estimates of price elasticities of public transit use [15], we estimated that transit ridership could increase between 6% and 14% in the short-term (<2 years), representing an additional 63,200 to 158,000 riders daily, and by as much as 26% in the long-term (>10 years). Although capacity of the present public transportation system in LAC exceeds demand by a factor of 3 to 1 [16], it is possible that the anticipated increases in passengers could lead to more crowding on some buses and trains.

**Table 1:** Estimates of decreases in public transit fare revenues for Los Angeles County transportation agencies if free transit passes were provided to all students.

Enrollment Status	Population Size *	Use of Public Transit ^†^ (%)	Average Number of Weekly Trips	Share of Total Weekly Fares Revenue ^‡^ (%)	Cumulative Cost Relative to Total Fare Revenue (%)
**Students**	**2,796,300**	**11.3**	**8.0**	**20.6**
Kindergarten through 8th grade	1,177,400	4.1	8.7	2.5	3
9th through 12th grade	642,500	15.4	7.7	4.5	7
College, trade, and other students	976,300	17.3	8.0	13.7	21
**Non-students**	**6,584,200**	**11.8**	**7.9**	**79.4**	**-**
**Total**	**9,380,500**	**11.7**	**7.9**	**100.0**	**-**

* Only ages 5 and older with student status information; estimated using weighted data from the 2010–2012 California Household Travel Survey (CHTS). **^†^** All self-reported public transit use in travel diary data. **^‡^** Assuming ratios of weekly paid fares across groups were the same as those estimated from the 2001 SCAG Travel Survey.

### 3.2. Potential Benefits

Benefits, if realized, could be significant for a variety of stakeholders (Table 2). With regard to school attendance, data supported that many students live far from their schools [17], that schools do not provide transportation for many students [10], and that many students rely on public transportation to get to school [18]. While many experts reported the positive impact of providing free transit passes on school attendance, we could only locate one evaluation of such a program, which showed limited impacts [19]. Therefore, to provide a rough estimate of potential impact, we used attendance data from LAUSD to estimate the instructional hours gained from a 1% and 5% decrease in unexcused absences.

With regard to contact with the juvenile justice system, data supported the high volume of fare evasion citations issued to youth, especially youth of color. While data were not available on the outcomes of these citations, protocols indicate that, if not diverted, citations could result in heavy fines (up to $250) or court appearances. Furthermore, data were available to support the negative outcomes associated with contact with the juvenile justice system (e.g., stress, drop out) [20,21].

Data suggest that the proposal could also result in increased income for schools. While shrinking budgets have caused many districts to reduce transportation services, in the 2011–2012 school year, LAC districts spent over $273 million providing transportation [10]. Providing free transit passes to students could result in school districts being able to redirect funds, providing that students currently served by school buses could use public transit (*i.e.*, that public transit is available and could meet students’ needs). Furthermore, as California schools are funded based on average attendance, we estimated that a 1% decrease in unexcused absences could results in an additional $125,000 per year in funding in LAUSD.

**Table 2:** Potential benefits of providing free transit passes to student in Los Angeles County, California.

Benefit	Key Findings
Increase in school attendance	• Three quarters of Los Angeles County school districts reported providing transportation for less than 10% of their students [10]. • 27% of students in Los Angeles County live more than 2 miles from their schools [17]. • Lack of affordable transportation is a frequently cited barrier to regular school attendance [9]. • For every 1% decrease in unexcused absences in Los Angeles Unified School District, students would receive 29,000 more instructional hours per year ^1^. • Students who attend school regularly are more likely to graduate, and have lower rates of incarceration, teen pregnancy, substance abuse, and chronic disease [22,23,24,25].
Decrease in contact with juvenile justice system	• The Los Angeles County Sheriff’s Department issued 9966 citations to youth (age <18 years) for fare evasion in 2012 ^2^. • Fare evasion citations can result in heavy fines (up to $250) or court appearances, which can lead to absences, missed work, and increased stress for youth and their parents. • A first-time court appearance during high school quadruples a student’s odds of dropping out [20,21].
Increase in available funds for schools	• For every 1% decrease in unexcused absences in Los Angeles Unified School District, schools would receive an additional $125,000 each year ^1^. • In the 2011–2012 school year, Los Angeles County school districts spent over $273 million providing transportation to students [10].
Healthier families and communities	• More students using public transportation could lead to fewer school-travel related injuries, including car fatalities and injuries related to unsafe neighborhood conditions [26,27]. • Free transit passes could save families $2.5 million per year on student transit passes ^3^. More disposable income could lead to less stress for families as well as increases in access to important resources such as healthy food, healthcare services, and opportunities for physical activity [28]. • More freedom and mobility could lead to youth accessing after-school activities, work, health care, and civic and religious events without being limited to their own neighborhoods [19,29]. • If 13,000 more students used public transportation (instead of driving or being driven to school), CO_2_ emissions could be reduced by 20.35 metric tons daily, the equivalent of saving over 2280 gallons of gasoline ^4^.

^1^ Calculated based on 2012–2013 administrative data from Los Angeles Unified School District for students in grades 6 through 12 and reimbursement rates for Average Daily Attendance in the State of California [30]; ^2^ Calculated based on administrative data from the Los Angeles County Sheriff’s Department for citations issued in 2012; ^3^ Calculated based on the cost of a monthly student transit pass ($24 per month) and the number of students that reported relying on public transportation to get to school in the Southern California Association of Governments Travel Survey (2001);^4^ Calculated based on the assumption that 50% of the additional short-term student transit riders would switch from using private car travel. Estimates generated using the EPA conversional tool [31].

Studies also supported the potential positive impacts of free student transit passes on decreases in injuries [26,27], increases in disposable income for families [28], increases in freedom and mobility for youth [19,29], and decreases in vehicle emissions, traffic congestion, and associated health benefits [32,33]. Unfortunately, due to limited data, the magnitude of these potential benefits was difficult to quantify. The level of uncertainty of all the potential benefits was described in the HIA’s technical appendix, which characterized the likelihood, magnitude, severity, and distribution of the impacts of the proposal based on clearly defined criteria [13].

### 3.3. Recommendations and Stakeholder Response

The goal of the HIA report was to summarize the best available data on the costs and potential impacts of providing free transit passes to students, as opposed to presenting specific arguments for or against the proposal. Because of the potentially large costs associated with the proposal and lack of data to quantify many of the benefits, recommendations were grounded in the need to better understand program impacts and tradeoffs. Specifically, recommendations included the need to: (a) explore key program and sustainability features, including possible sources of funding through partnerships between transportation agencies and school districts; (b) consider tradeoffs and find ways to operationalize the program so as to maximize positive impacts and minimize costs (e.g., establish eligibility criteria for free passes, expand existing transportation subsidization programs); (c) collect additional program-specific data, for example, though a pilot study, to more precisely evaluate the potential impacts of the program; and (d) continue to engage transit agencies in discussions moving forward.

Presentations made to both community-based organizations and governmental agencies were well received. DPH received feedback that the HIA provided a useful, comprehensive synthesis of the potential costs and benefits, helping to contribute to ongoing dialogue related to transit pricing in the region. Despite the inability to quantify many of the potential benefits, the report represented the most comprehensive synthesis of information of the topic. Decision-makers noted the alignment between estimates of revenue and ridership derived in the report and those provided to them by MTA, boosting confidence in the results.

Because of budget shortfalls, in May 2014, the MTA Board of Directors passes a resolution to increase fares on busses and trains for all groups of riders, except kindergarten through 12th grade students [34]. While many stakeholder efforts and opinions contributed to this decision, data from the HIA was used within a LAC Board of Supervisors resolution (passed in conjunction with the fare increase), that expressed the need to make meaningful attempts to address affordable transportation as a barrier to regular school attendance and decrease the criminalization of fare evasion amongst youth riders [35].

## 4. Lessons Learned

### 4.1. A Broad Conceptualization of the Potential Policy Impacts Brings Opportunities and Challenges

By taking a broad perspective on the potential impacts of the proposal, DPH was able to engage a wide-range of partners affected by student transportation, including schools, law enforcement (e.g., Sheriff, Los Angeles School Police), juvenile justice agencies (e.g., probation, juvenile courts), transportation officials (e.g., MTA), child and family welfare agencies, and environmental groups. The HIA expanded understanding that the resolution might affect not only educational attainment and criminalization of youth, but also health, safety and the environment. Incorporating the perspectives of diverse partners carried many benefits, including increased dialogue about the interconnectivity among different agencies’ goals. Decision-makers commented on the helpfulness of the report’s pathway diagram (Figure 1), which illustrated the connection between the resolution’s short and long term impacts. Additionally, describing the impact of the resolution on diverse outcomes widened the potential base of support (e.g., funding sources) for the proposed policy.

Working with a diverse group of stakeholders also increased the complexity of the process. Benefits of the proposal were found to be distributed broadly to multiple stakeholders, but the costs of providing free transit passes (at least initially) fall disproportionately on transportation agencies, which are unlikely to benefit in the short term. Community partners framed the issue of student transportation as one of social justice, while transportation partners maintained a stronger focus on financial and operational implications. To help address this challenge, DPH maintained open communication with all partners and provided an opportunity for all groups to contribute data to the HIA, including both MTA budget estimates as well as data collected by community-based partners about student experiences with public transportation.

Even among community-based partners, perspectives differed. Community-based organizations differed in their perspectives on the best use of public transportation funds (e.g., whether to focus on bus or rail infrastructure). Opinions also differed on the ways in which the resolution should be operationalized—e.g., the group of students who should receive free passes, when the passes should be valid. The call for universal provision of passes, without a trip-purpose or time limitation, was intended to decrease stigma associated with public assistance to low-income students and maximize participation in discretionary activities, such as after-school and cultural programming. Employing the use of multiple scenarios (e.g., providing passes to all students *versus* only low income students) helped illustrate the trade-offs between decisions of who should receive passes.

### 4.2. Need for Data to Reliably Estimate the Potential Impacts on Sub-Populations

The transit pass resolution was seen by many as one “common sense” solution to the problem of school attendance. Many school districts and social service agencies spoke of families’ and students’ challenges in paying for public transportation and the great value of the proposal. However, when it came to quantifying the potential impact of the resolution on improving school attendance, limited data were available. Although other jurisdictions in the U.S. (e.g., San Francisco, Portland, New York City) have implemented free transit pass programs, few have evaluated the impact of such efforts or compared the benefits to programmatic costs.

HIA estimates of the impact of a program or policy decision are only as strong as the data that support them. In our assessment, population-level data were of limited utility because information about transportation and school attendance behaviors was not co-located or able to be linked. Frequently, agency data systems collect data on a narrowly defined set of indicators (*i.e.*, those directly relevant to their programs). While this facilitates internal monitoring and evaluation, it limits the ability to examine the association between policy and program decisions and other outcomes of interest.

HIAs frequently need to be completed on short timelines; timelines that do not allow for primary data collection. With complex issues (such as school attendance) that are influenced by a web of inter-related factors (e.g., transportation, family structure, health, school climate) it is difficult to assess any single policy change. Many have noted data availability and quality as challenges to HIA practice [6,8]. To better inform decision-making, we need more comprehensive, longitudinal data systems as well as dynamic models that can synthesize data from multiple sources and facilitate assessment of multiple policy and program options.

### 4.3. HIA is One Component of a Comprehensive Policy Assessment Strategy

Partners were grateful to have estimates of the proposal’s potential costs, because questions on short-term budget impacts were a central concern to decision-makers. However, HIAs traditionally do not include cost assessments because of limited scope or data. The lack of inclusion of cost data—often a key component of decision making—may hinder the usefulness of HIA results. It is unlikely that DPH’s assessment would have received such a positive reaction from partners without the cost estimates.

The HIA was seen as a good first step in informing a policy debate; however, the results raised many additional questions related to the feasibility of the policy. Stakeholders wanted more information related to expenditures, including the current level of spending on transportation by LAC school districts and County departments (e.g., Department of Children and Family Services) as well as examples of funding models used by other jurisdictions. While DPH strove to address some of these issues in the report *Addendum*, a detailed assessment of the “best practices” to implement and fund the policy was beyond the scope of the HIA.

HIAs represent one tool in the toolbox of cross-sector policy work. While HIAs generally succeed in outlining potential effects and equity consequences of a discrete policy option, decisions are often made using additional criteria such as political acceptability, feasibility and financial constraints. Consequently, public health professionals working with decision-makers in other sectors should expect a HIA to spur additional lines of inquiry. Indeed, a well-conducted HIA may provide an opportunity to engage in additional collaborative projects, such as feasibility assessments or evaluation of policy-mandated programs. Being responsive to partner requests has helped DPH to strengthen its relationships with the ECC member agencies.

### 4.4. Public Health’s Role in Cross-Sector Work Is Not Universally Acknowledged

Despite mounting evidence of the connection between health and decisions made in other sectors and the momentum to adopt a HiAP approach in public health practice [1], the nascent role of public health in cross-sector work remains underappreciated and underutilized. Understanding the reasons for DPH’s involvement in the transit project and the conceptualization of student attendance as a public health issue varied widely among the partner agencies.

Criticism from within the public health community emerged from the lack of a core focus on “health outcomes” in the HIA results. Because the assessment was driven by the primary concerns of non-health stakeholders (*i.e.*, school attendance and youth contact with the juvenile justice system), the results were framed around these outcomes. Furthermore, the short timeline and lack of available quantitative data only allowed DPH to quantify the short-term benefits. While the connection between these short term impacts and longer term health outcomes (e.g., violence, teen pregnancy, substance abuse, life expectancy) was discussed in the HIA report, the lack of health outcomes “front and center” raised concern among some partners as to why public health was involved in the issue at all.

DPH took many steps to address these challenges. Communicating and framing the HIA report to highlight the well-established connection between educational attainment and health helped justify the assessment. Logic pathways were useful in illustrating the relationship between transportation policy decisions and subsequent academic, social, and health outcomes. While DPH strengthened understanding among partners, the challenges that it faced during the HIA suggest that additional efforts to educate both health and non-health partners about public health’s role in public policy and social welfare may be warranted. Better messaging about the interconnectivity between agency goals may facilitate the use of HIA in other cross-sector collaborative efforts.

## 5. Conclusions

The HIA provided a concrete mechanism to engage a wide variety of stakeholders and synthesize information about the potential impacts of providing free transit passes to students in LAC. To date, the HIA has been well received by partners and has been described as a valuable tool in helping them develop cross-sector messages, operationalize the logistics of the transit proposal, and communicate with decision-makers about the proposed program. An important outcome of the HIA was the opportunity for DPH to engage in cross-sector dialogue with non-traditional, non-health partners. The assessment and cross-sector engagement enhanced the credibility of the DPH as an unbiased source for policy assessment and program evaluation. This should pay dividends in future policy work and has already provided a springboard for launching additional collaborative projects within the county.

Lessons learned in conducting the transit HIA helped identify key opportunities for public health to enhance its cross-sector portfolio. Specifically, best practices for stakeholder engagement, establishing more comprehensive data systems and models, use of HIA and complementary policy analysis tools, and better messaging of policy implications all represent critical components of an evolving infrastructure that support cross-sector collaboration and policy development. As public health moves forward in implementing a HiAP agenda, further development of these components will be vital for laying the necessary foundation that can inform policy development and achieve meaningful cross-sector engagement.
